# Relating professionalism and conscientiousness to develop an objective, scalar, proxy measure of professionalism in anaesthetic trainees

**DOI:** 10.1186/s12909-017-0891-6

**Published:** 2017-03-01

**Authors:** M. A. Sawdon, K. Whitehouse, G. M. Finn, J. C. McLachlan, D. Murray

**Affiliations:** 1 School of Medicine, Pharmacy and Health, Durham University, Queen’s Campus, University Boulevard, Thornaby, Stockton-on-Tees TS17 6BH UK; 2 Royal Victoria Infirmary, Newcastle-upon-Tyne, UK; 3 Hull York Medical School, York, UK; 4 Durham University, Stockton-on-Tees, UK; 5 James Cook University Hospital, Middlesbrough, UK

**Keywords:** Assessment, Professionalism, Conscientiousness, Anaesthetists

## Abstract

**Background:**

The concept of professionalism is complex and subjective and relies on expert judgements. Currently, there are no existing objective measures of professionalism in anaesthesia. However, it is possible that at least some elements of professionalism may be indicated by objective measures. A number of studies have suggested that conscientiousness as a trait is a significant contributor to professionalism.

**Methods:**

A ‘Conscientiousness Index’ (CI) was developed by collation of routinely collected data from tasks expected to be carried out by anaesthetic trainees such as punctual submission of holiday and ‘not-on-call’ requests, attendance at audit meetings, timely submission of completed appraisal documentation and sickness/absence notifications. The CI consists of a sum of points deducted from a baseline of 50 for non-completion of these objective and measurable behaviours related to conscientiousness. This was correlated with consultants’ formal and informal subjective measures of professionalism in those trainees.

Informal, subjective measures of professionalism consisted of a ‘Professionalism Index’ (PI). The PI consisted of a score developed from consultants’ expert, subjective views of professionalism for those trainees. Formal, subjective measures of professionalism consisted of a score derived from comments made by consultants in College Tutor feedback forms on their views on the professionalism of those trainees (College Tutor feedback; CT). The PI and CT scores were correlated against the CI using a Pearson or Spearman correlation coefficient.

**Results:**

There was a negative, but not statistically significant, relationship between the CI and formal, subjective measures of professionalism; CT scores (*r* = -0.341, p = 0.06), but no correlation between CI and consultants informal views of trainees’ professionalism; the PI scores (*r*_*s*_ = -0.059, *p* = 0.759).

**Conclusions:**

This may be due the ‘failure to fail’ phenomenon due to the high stakes nature of raising concerns of professionalism in postgraduate healthcare professionals or may be that the precision of the tool may be insufficient to distinguish between trainees who generally show highly professional behaviour. Future development of the tool may need to include more of the sub-facets of conscientiousness. Independently of a relationship with the construct of professionalism, a measure of conscientiousness might be of interest to future employers.

## Background

Professionalism is a complex construct, with many definitions and attributes [1], but one which is accepted to be important. Fitness to practice cases often involve what is described as ‘unprofessional behaviour’ or a ‘lack of professionalism’. Studies have shown a link between unprofessional behaviour in training and subsequent disciplinary action in later practice [2, 3]. In parallel with other specialties, there have been attempts to define professionalism in anaesthesia in addition to attempts to better understand how professionalism might be better taught and assessed within anaesthesia [4–9]. Currently, there are no existing objective measures of professionalism in anaesthesia, and assessment of professionalism relies on subjective, expert judgements. Subjective measures have inherent problems with reliability, requiring repeated measures which are not always possible in order to ensure a consistent score.

The measures of professionalism discussed by Papadakis et al. [2, 3] essentially involve a subjective rating or judgment. However, it is possible that at least some elements of professionalism may be indicated by objective measures. A number of studies have suggested that the trait of conscientiousness is a significant contributor to professionalism [10]. Conscientiousness may be indicated by defining occasions on which the trainee might carry out actions which can be reasonably expected of them (such as attending compulsory training sessions and completing essential administrative documentation) and recording whether those actions have been carried out. It has been suggested that objective measures of this kind have the potential to be used to assess professionalism in anaesthetic trainees [11].

Previous studies have demonstrated that measurement of such activities - codified as a ‘Conscientiousness Index’ (CI) – positively co-distributes with the construct of professionalism as determined by experienced educational staff [12], and by peers [13] in the preclinical years of an undergraduate medical programme. These results have been repeated in undergraduate medical students in their clinical years in another country [14]. A key aspect of building a CI is that the data included is generally already being collected for other purposes, and only centralisation is required, meaning the data is inexpensive to collect. In addition, it is determined over many occasions rather than a few observed sessions.

The CI instrument has already been adapted for use with paramedics in training; with results showing the CI significantly correlates with the trainers’ score of trainees’ professionalism [15], and is under evaluation for use in other specialties. This indicates it has credibility in health care settings other than undergraduate medical education. As far as we are aware this is the first such attempt to explore the use of a ‘Conscientiousness Index’ in residency training.

Conscientiousness may be a part of professionalism, and independently may well be predictive of performance in other areas. It is already well established that conscientiousness measured through personal qualities tests has predictive validity for job performance in general [16]. The advantage of McLachlan’s approach is that it relies on direct observation of behaviour, rather than subjective or self-report instruments [12].

The aim of this study was to explore the relationship of a ‘Conscientiousness Index’ (CI) in anaesthetic trainees with current, subjective, measures of professionalism in this specialty.

## Methods

The project gained local NHS Trust R&D and Durham University, School of Medicine and Health Ethics Sub- Committee approval in May 2012.

As this study was the first of its kind in a postgraduate cohort we did not know if previous effect sizes seen in our CI studies in undergraduate students [13] would be appropriate to use to calculate a minimum sample size for this study and thus we were unable to carry out a power analysis. In addition, we did not know how many trainee anaesthetists would volunteer to take part and so aimed to recruit as many as possible on rotation at one local hospital. All 52 anaesthetic trainees at that hospital were invited to take part and 32 trainees volunteered and consented to participate in the study during 2012–2013. The identities of trainees were anonymised by allocation of a unique code to each trainee. The data was collated by School of Anaesthesia administrative staff and passed on to the research team for analysis.

All CI data was obtained from information that is already available to administrative and clinical staff within the School of Anaesthesia so consent for its collection was not required [17]. However, consent was gained for it to be passed on, in an anonymised form, to the research team. The consent process stressed that the information was collated for research purposes and that their CI score would have no bearing on their workplace assessments or progression through the anaesthetic training programme.

All trainees at the study hospital are routinely regularly assessed by over 50 anaesthetic consultants as part of their training. The results of this study did not have a bearing on trainees’ progression, and nor indeed could it since CI scores were not passed on to those assessing them. The ultimate decision about a trainee's progression through the training programme is made at the Annual Review of Competence Progression (ARCP) meeting. However, CI scores were not made available to this panel either.

There are already mechanisms at the hospital in question and throughout the local Deanery to detect and deal with trainees who exhibit unprofessional or unacceptable behaviour. These have been developed over time and are currently considered robust, and do not include the CI. The aim of this study was to explore the relationship of the CI score with existing assessments of professionalism.

### Development of the Conscientiousness Index

As the Conscientiousness Index (CI) should be comprised of information which is easily available to the training provider, it is necessarily particular to the organisation in which it is being used. As such, its relationship with professionalism would need to be validated in these new contexts, and this is the purpose of this study. After initial consultations with senior anaesthetists and administrative staff in the School of Anaesthesia at the study hospital, appropriate sources of objective data were identified. In order to be included, data had to be easily and readily available to administrative staff, and could be collected on anaesthetists at all stages of training, from Core to Specialty Training. From this information the components of the Conscientiousness Index were agreed. In line with other studies on the Conscientiousness Index [12, 14] trainees were awarded a baseline of 50 points to avoid negative scores at the end of the study. Due to the nature of the data collected (i.e., the behaviours were “omissions”) it was more appropriate to deduct points for non-completion rather than award points for completion; e.g., not informing the department of an unplanned absence. The CI is thus a sum of points deducted from a baseline of 50 for non-completion of objective and measurable behaviours related to conscientiousness, and calculated as a percentage of the overall maximum CI score attained at the end of the study. Subjective measures were not included. Table 1 shows the list of components that make up the CI for trainee anaesthetists, and the amount of points deducted for non-completion of each. The number of points deducted was related to the perceived “seriousness” of the omission.

**Table 1:** Components and scoring of the Conscientiousness Index (CI). All trainees start with 50 points (in line with other work on CI [14]) this prevents negative scores occurring

Component	Notes	CI Points
Sickness/absence	If the trainee was off sick or absent and did not let department know	−10 for each occasion
Audit meeting attendance	Percentage of audit meetings the trainee could have attended but missed	The percentage was divided by 5 to reduce the weighting of this component on the overall CI score. This value was then deducted from the total CI score
Appraisal documentation	Did they submit appraisal documentation within requested timescale? And complete?	0 if all submitted and on time -5 if not submitted on time or incomplete -10 if not submitted on time AND incomplete
Short notice requests	Requested change in rota or ‘not-on call’ or holiday request less than 6 weeks in advance (School policy states requests should be made more than 6 weeks in advance of any requested change)	Sliding scale: Request made more than 6 weeks in advance; 0 points 5–6 weeks in advance -1 4–5 weeks in advance -2 3–4 weeks in advance -3 2–3 weeks in advance -4 1–2 weeks in advance -5 Less than 1 week in advance -6

Individual data points were reviewed on a case by case basis for justifiable reasons for non-completion of the event. For instance, if a short notice request was due to unavoidable factors outside the trainee’s control, it was not counted against them.

### Validity measures

#### Concurrent validity of the Conscientiousness Index with workplace based assessment of professionalism; The ‘College Tutor’ score

Concurrent validity refers to the agreement between variables which purport to measure the same or related constructs. The CI measures the trait of conscientiousness, which we hypothesise might be part of the construct of professionalism. Parts of the existing workplace based assessment (trainees’ College Tutor feedback) are intended to measure professionalism in practice, and so the relationship between the two was explored.

All trainees receive regular feedback on their progression and professionalism from a pool of over 50 consultant anaesthetists who work with the trainees over the course of their rotation. The College Tutor collates the feedback and generates a report on the trainee. Aspects such as clinical skills, personal characteristics and confidence are commented on for their appropriateness to training grade. Reports were available for all but one anaesthetic trainee participating in this study. The free text written by the consultants on the trainee’s behaviour within these reports was scored by the researchers as follows; any positive comment made was scored +2, any ‘excellent’ (or related words, e.g., ‘outstanding’, ‘brilliant’) comment +3, any ‘no concerns’ comment +1, any negative comment scored -4.

A ‘CT’ (College Tutor) score was calculated by summing these scores and dividing by the number of consultants exposed to that trainee (i.e., did or could have commented, as indicated on the feedback report). This was to ‘normalise’ the data between trainees receiving different numbers of consultants’ feedback.

#### Concurrent validity of the Conscientiousness Index with senior anaesthetists’ expert judgements on trainees’ professionalism; The ‘Professionalism Index’

A randomised list was compiled of participating trainees’ names and, isolated from the knowledge of their CI scores, the list was given to senior (Consultant) anaesthetists responsible for guidance of these trainees (and thus having some knowledge of them) and they were asked to express an expert judgement regarding the trainees’ professionalism by choosing, for each trainee, one option from this list:

- I am happy with the professionalism shown by this trainee.
- I have some concerns with the professionalism of this trainee.
- I do not know this trainee well enough to comment.

In our discussions with stakeholders, it was clear that understandings of the construct of professionalism are complex and variable from individual to individual. We therefore decided to use this very simple rating scale, in line with our previously published work [12].

A ‘Professionalism Index’ (PI) for 29 of the 32 trainees (some trainees were scored as ‘*I do not know this trainee well enough to comment’* by Consultants) was then compiled from the results of this with the ‘happy’ scores expressed as a percentage of the total ‘happy’ and ‘concerns’ scores. This was to normalise the data and was slightly different to earlier studies whereby the PI was calculated as the ‘Happy’ scores minus the ‘Concern’ scores [12, 14] as in this study there were different numbers of consultants scoring the participants (from 2 for some participants, to 20 for others).

### Statistical analysis

Each trainee’s data (CI, PI and CT scores) was entered into IBM SPSS Statistics Developer 20. Tests of normality were carried out (Kolmogarov-Smirnov test); the CI (D [32] = 0.143, *p* = 0.095) and CT data (D [31] = 0.147, *p* = 0.084) were normally distributed, but the PI scores were not (D [29] = 0.430, *p* < .001). Any correlation between the CI and PI scores for each trainee was thus statistically explored using the nonparametric Spearman Rank correlation coefficient, whereas any correlation between CI and the CT was explored using a Pearson correlation.

## Results

### The Conscientiousness Index (CI)

**Fig. 1:**
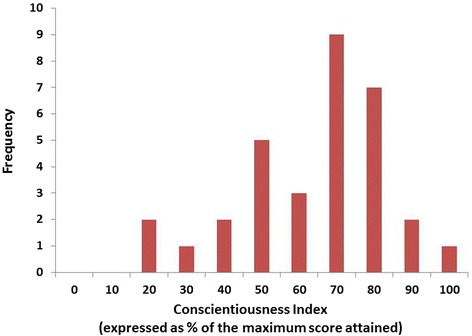
The Conscientiousness Index scores in trainee anaesthetists. The frequency distribution of Conscientiousness Index scores shown as percentages of the maximum score attained for trainee anaesthetists (*n* = 32) at one hospital during 2012–2013

Figure 1 shows the frequency distribution for the CI scores for the 32 trainee anaesthetists in the study (21 males, 11 females). The range of ‘raw’ CI scores was 10–47 (from the baseline of 50 awarded to each trainee). The range of CI scores expressed as a percentage of the maximum score attained was 21–100%. The mean CI score (expressed as a percentage of the maximum score attained) is 68% and SD 19.8% (Table 2).

**Table 2:** Descriptive statistics; range of scores, their mean and standard deviations (SD) for the Conscientiousness Index (CI) and College Tutor Feedback (CT). Professionalism Index (PI) is expressed as the median and interquartile range as this data did not follow a normal distribution. *n* = number of participants data was collected on in each group (from the total of 32 in the study)

Measure	Score range	Mean	SD	*n*
CI	21–100%	67.6%	19.8%	32
PI	73–100%	100% (median)	8.5 (IQR)	29
CT	−0.2–2.2	1.1	0.5	31

### Concurrent validity of CI with workplace based assessment: the College Tutor (CT) score

**Fig. 2:**
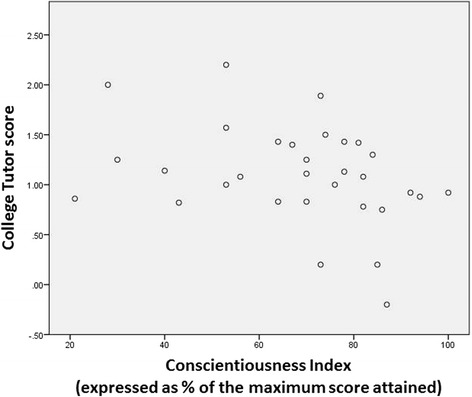
Scatter plot showing the relationship between the Conscientiousness Index (CI) expressed as a percentage of the maximum score attained and College Tutor feedback scores

The range of scores was -0.2 to 2.2, with a mean of 1.1 and SD 0.5 (Table 2). There was a negative, but not statistically significant, relationship between CI and the College Tutor feedback score (see Fig. 2 and Table 3; *r* = -0.341, *p* = 0.06).

**Table 3:** Results of statistical comparisons for the Conscientiousness Index scores (CI), the Professionalism Index scores (PI) and the College Tutor feedback scores (CT). See text for a description of each item

Correlation	Pearson (r)	*p* value	Spearman (*r*_s_)
CI vs PI		0.759	−0.059
CI vs CT	−0.341	0.06	
CT vs PI		0.842	−0.04

### Concurrent validity with experts’ judgements of professionalism; the ‘Professionalism Index’ (PI)

PI scores ranged from 73 to 100% (median 100%, interquartile range 8.5; Table 2). No correlation was apparent between the CI and PI scores for each trainee (Table 3; *r*_s_ = -0.059, *p* = 0.759).

## Discussion

A Conscientiousness Index (CI) was successfully developed for anaesthetic trainees (the spread of scores and descriptive statistics compare with those in the literature [12, 14]). However, this initial exploration in this particular group of healthcare professionals has shown no correlation between the objective measure of conscientiousness (CI) and consultants’ expert subjective views of professionalism as measured for this study by calculation of what we termed the ‘Professionalism Index’ (PI). There was a negative, but not statistically significant, relationship (Table 3) with the CI and the coded subjective free text comments on trainee anaesthetists’ professionalism by their seniors; the College Tutor feedback score (CT). The fact that this is negative means that the senior anaesthetists responsible for these trainees’ assessments appear to rate trainees’ professionalism high (in formally assessed measures as part of the trainees’ ongoing assessment for progression) whilst their objective Conscientiousness Index scores are on the lower end of the scale (Fig. 2). However, the College Tutor feedback system did not seem to specifically ask about traits related to conscientiousness and this may have been one of the confounding factors in scoring professionalism using the College Tutor feedback system. The positive and negative comments given by the senior consultants about their trainees may often be associated with trainee likeability and therefore would not necessarily reflect on professionalism/conscientiousness.

However, the lack of a correlation between the measured conscientiousness and consultants views of professionalism in the same trainees may be due to the ‘failure to fail’ phenomenon [18, 19] as a result of the high stakes nature of raising concerns about professionalism in postgraduate healthcare professionals. This problem is cited as the “single most important problem with evaluation” in one institute [20]. Reasons for failing to fail medical students and residents have been given by faculty clinicians as lack of adequate documentation, lack of knowledge of what to document, the potential consequences to the reporting clinician of subsequent appeals, and perceived lack of a remediation process [19].

Interestingly, there was no correlation between the formal assessments of trainees, the College Tutor (CT) score, and the informal (for the purposes of this study) assessment, the Professionalism Index (PI) scores, which leads to the question, are they assessing the same thing? The CT reports are generated from consultants’ assessments of different aspects of a trainee’s work including areas associated with professionalism, so a correlation might be expected. Thus the lack thereof may be further evidence of the failure to fail phenomenon when the stakes are high [18]; the Professionalism Index assessment did not have any bearing on the trainees’ yearly assessments in contrast to the College Tutor report which forms part of a trainees’ ongoing assessment for progression. Alternatively, the relationship between conscientiousness and professionalism apparent in other settings may not apply at higher levels of medical training.

The Conscientiousness Index was tailored to the anaesthetic department environment after discussion with several consultant anaesthetists, but it may be that we did not include a sufficient range of objective behaviours. Previous work on the CI [12, 14, 15] has included data such as attendance, punctuality (e.g., punctual submission of written work and/or punctual arrival on training days) and completion of evaluation questionnaires. Although this study did collect data on attendance at audit meetings the weighting of this item in the CI was scaled down (see Table 1) as it was thought by senior anesthetists that this was not particularly important relative to other conscientious acts and should not have too much influence on the final CI score. Punctuality was also captured by short notice requests. However data on whether trainees took part in evaluations (e.g., of teaching modules) was not used as this data was not routinely collected. Previous analyses has shown taking part in such evaluation to be the strongest correlator to the overall CI [21]. Research commissioned by the Health and Care Professions Council (HCPC) to investigate professionalism and conscientiousness in paramedics found differences in CI results between organisations and concluded that this was likely to be due to differences in the amount of data collected regarding opportunities to display conscientiousness; more data points led to stronger relationships between CI and trainers’ views of their professionalism [15]. Therefore we may have collected the right type of data to capture an accurate view of conscientious behaviour but we may not have captured this over sufficient opportunities for anaesthetists to display such behaviour. Data was collected on each trainee in the study for only 6 months whilst on rotation at that hospital. This is in contrast to previous work where data was collected over a full academic year [12, 14]. Although the original study showed the CI to be stable when performance over the first half of the year was compared with performance over the second half [12], it may be that in this study consultants did not get the chance to spend enough time with individual trainees over the course of their rotation to make a reliable judgement about their professionalism. There may also be fewer opportunities to assess professionalism over those 6 months.

As the participants in this study were self-selected volunteers, their willingness for their conscientiousness to be monitored for the purpose of research during their rotation may indicate that these are amongst the more highly conscientious of the anaesthetic trainees. The original study collected data on all students to avoid students ‘faking it’, especially as some of the points available in that study could be gained from volunteering to help out during extra-curricular events [12]. In addition to this participants were aware of the type of data that we were collecting and so may have made a concerted effort to be more diligent over carrying out more administrative tasks during this time (although if they can ‘fake it’ for the whole rotation does that make them conscientious anyway?). It was a requirement of the ethics review that the participants were informed of the type of data being collected on them and thus the following sentence was included in the participant information sheet; “*[The CI] is likely to include several components such as punctual submission of holiday requests and completed workplace training assessments*.”

The original work on CI [12–14] was carried out in a medical undergraduate population where explicit student consent was not required or sought. There are a number of assessment and application hurdles between medical school and starting anaesthetic training. The numbers of anaesthetic trainees deemed ‘unconscientious’ or ‘unprofessional’ may be significantly smaller than in the undergraduate population, given the barriers that have been overcome, and earlier opportunities to intervene if trainees show unprofessional behaviour. Since this is our first study in post graduate environments we did not know if the effect size we achieved in our previous studies on the CI [13] would be sufficient to power this study, or indeed how many participants we would obtain as volunteers. The fact that we did not observe a relationship might suggest there is a possible upper limit for the effect size for future studies on CI in the postgraduate environment. We suggest a much larger sample size would be needed to detect any differences in conscientiousness or professionalism in such a highly conscientious group.

Trainees may be reluctant to participate in such studies due to perceived repercussions of one’s conscientiousness being observed, despite reassurances in the information sheet that there would be no repercussions and all data would be anonymised. Different results may be found with an increase in sample size, especially if trainees are not require to provide explicit consent, and this warrants further investigation if we are to be confident that trainee anaesthetists’ professionalism is being adequately assessed. However, the spread of professionalism may have been too small in this cohort of trainees, and the precision of the CI tool may be insufficient to distinguish between trainees who generally show highly professional behaviour.

### Feasibility and utility

There were issues around data collection for this study and this has been reported in other studies involving measuring conscientious behaviour in a postgraduate healthcare setting [15]. For such a tool to be useful, it ideally needs to use readily collectable data that simply needs collating. The data collected in this study was derived from several sources and involved several different people, leading to logistical issues. Consequently some of the original data that was planned for collection could not be accessed. As a result, many of the objective behaviours measured related to personal organisation, whereas there are other behavioral domains within the trait of conscientiousness. Conscientiousness, as a higher-order personality domain, can be divided into 6 lower-level facets; orderliness, dutifulness, achievement-striving, self-discipline, cautiousness, and self-efficacy, [22]. Perhaps we have only captured the first one or two of these. It is perhaps worth noting here that the CI has previously been shown to significantly correlate with all of those facets except self-efficacy [23]. Therefore future development of this tool may need to be designed to include items that sample each of these facets.

A CI that uses a greater number and wider range of components would give such a scale more granularity and thus may be more accurate, but may have its own ‘costs’ in terms of establishing a data collection system. In previous studies [12, 14] the CI has been shown to be stable, and ‘cost’ (in terms of staff time) was low (although acceptability by the students may have been questioned! [24]). However these studies were in the undergraduate setting. So there has to be a tradeoff between the feasibility, reliability and validity of the assessment tool.

## Conclusions

In this study, we did not observe a relationship between a measure of conscientiousness and a measure of professionalism. This may be due to variance in reporting either conscientiousness or professionalism, or a true lack of a relationship between conscientiousness and professionalism in this setting. We are aware that in selection decisions, measures of conscientiousness might be viewed as desirable, but between two candidates of equal clinical skill, we do not think this is necessarily a bad thing. Therefore, independently of a relationship with the construct of professionalism, a measure of conscientiousness might be of interest to future employers.

## Abbreviations

ARCP: Annual review of competence progression
CI: Conscientiousness Index
CT: College tutor feedback score
PI: Professionalism index
R&D: Research & development
